# Delineating excess comorbidities in idiopathic pulmonary fibrosis: an observational study

**DOI:** 10.1186/s12931-024-02875-2

**Published:** 2024-06-19

**Authors:** Burcu Ozaltin, Robert Chapman, Muhammad Qummer Ul Arfeen, Natalie Fitzpatick, Harry Hemingway, Kenan Direk, Joseph Jacob

**Affiliations:** 1 Satsuma Lab, Centre for Medical Image Computing, UCL, London, UK; 2 UCL Respiratory, UCL, London, UK; 3 UCL Institute of Health Informatics, UCL, London, UK; 4 Imperial Clinical Trials Unit, Imperial College London, London, UK

**Keywords:** Idiopathic pulmonary fibrosis, Chronic obstructive pulmonary disease, Comorbidities

## Abstract

**Background:**

Our study examined whether prevalent and incident comorbidities are increased in idiopathic pulmonary fibrosis (IPF) patients when compared to matched chronic obstructive pulmonary disease (COPD) patients and control subjects without IPF or COPD.

**Methods:**

IPF and age, gender and smoking matched COPD patients, diagnosed between 01/01/1997 and 01/01/2019 were identified from the Clinical Practice Research Datalink GOLD database multiple registrations cohort at the first date an ICD-10 or read code mentioned IPF/COPD. A control cohort comprised age, gender and pack-year smoking matched subjects without IPF or COPD. Prevalent (prior to IPF/COPD diagnosis) and incident (after IPF/COPD diagnosis) comorbidities were examined. Group differences were estimated using a t-test. Mortality relationships were examined using multivariable Cox proportional hazards adjusted for patient age, gender and smoking status.

**Results:**

Across 3055 IPF patients, 38% had 3 or more prevalent comorbidities versus 32% of COPD patients and 21% of matched control subjects. Survival time reduced as the number of comorbidities in an individual increased (*p* < 0.0001).

In IPF, prevalent heart failure (Hazard ratio [HR] = 1.62, 95% Confidence Interval [CI]: 1.43–1.84, *p* < 0.001), chronic kidney disease (HR = 1.27, 95%CI: 1.10–1.47, *p* = 0.001), cerebrovascular disease (HR = 1.18, 95%CI: 1.02–1.35, *p* = 0.02), abdominal and peripheral vascular disease (HR = 1.29, 95%CI: 1.09–1.50, *p* = 0.003) independently associated with reduced survival. Key comorbidities showed increased incidence in IPF (versus COPD) 7–10 years prior to IPF diagnosis.

**Interpretation:**

The mortality impact of excessive prevalent comorbidities in IPF versus COPD and smoking matched controls suggests that multiorgan mechanisms of injury need elucidation in patients that develop IPF.

**Supplementary Information:**

The online version contains supplementary material available at 10.1186/s12931-024-02875-2.

## Introduction

Idiopathic pulmonary fibrosis (IPF) is a rare and progressive form of interstitial lung disease that is increasing in incidence [1]. IPF demonstrates a varied and unpredictable clinical course [2], with an average survival time of 3–5 years from diagnosis [3, 4]. Patients diagnosed with IPF are typically over 50 years of age, predominantly male and typically have a history of tobacco use [5]. Though there are no curative treatment options for IPF, two molecular therapies (nintedanib and pirfenidone) have demonstrated efficacy in reducing disease progression [6, 7] and improving survival [8]. The advent of therapies that curtail the progression of IPF, has allowed a focus on the comorbidities that may be found in patients with IPF and an assessment of their impact on patient survival.

Single centre cohort studies have shown that comorbidity is common in IPF, reported in up to 89% of patients [9, 10]. The most frequently reported comorbidities seen in IPF include cardiovascular disease [11], cardiac failure [12], pulmonary hypertension [13], emphysema [14], lung cancer [15], gastro-oesophageal reflux disease [16] and depression [17]. The presence of comorbidities has a negative effect on mortality in IPF and survival has been shown to decrease as the number of comorbidities in an individual increases [9, 18–20]. Up to a quarter of patients with IPF reportedly die from non-respiratory causes of death [21] which may be explained by coexisting comorbidities.

Chronic obstructive pulmonary disease (COPD) is another chronic respiratory disease that primarily affects smokers, and which has been associated with multiple comorbidities. The high prevalence of multimorbidity found in patients with COPD is thought to be a consequence of chronic pulmonary and systemic inflammation [22, 23]. The frequency of comorbidities in COPD has led to a view of COPD as a disease of multimorbidity [24], and a great deal of research has been undertaken to develop prognostic tools in COPD based on the burden of comorbidity [25].

The increased burden of comorbidity previously identified in IPF and COPD may have multiple driving factors, such as cigarette smoking, chronic inflammation or accelerated ageing. Our study focussed on understanding the magnitude of differences in comorbidity prevalence between patients with IPF and those with COPD. By utilising primary healthcare records, we gathered data on the occurrence and mortality impact of prevalent (diagnosed on or before the date of IPF and COPD diagnosis) and incident (those diagnosed after the index period) comorbidities. We compared results in an IPF population, with age, gender, smoking (pack-year) matched COPD patients and age, gender, smoking (pack-year) matched controls without a diagnosis of IPF or COPD.

## Methods

### Data source

We used pseudonymised patient-level, electronic health care records from the Clinical Practice Research Datalink (CPRD) GOLD database (January 2019). A detailed description of this resource is described elsewhere [26]. The study was approved by the MHRA (UK) Independent Scientific Advisory Committee [18_291], under Sect. 251 (NHS Social Care Act 2006).

### Study population

We established a cohort of IPF patients diagnosed between 1st January 1997 and 1^st^ January 2019 from the patients with multiple registrations cohort. The date of the first attendance where an read code (medical code) mentioned IPF was taken as the date of diagnosis. We excluded IPF patients where the date of the initial attendance with an read code for IPF was on the date of death, as well as IPF patients whose death date was less than 30 days after having been diagnosed (Supplementary Fig. 1). IPF patients with a history of a rheumatological disease were excluded from the analyses. COPD patients were 1:1 matched with IPF patients according to patient age, gender, smoking history (never versus ever), all at the time of the first COPD diagnosis. A further control population comprised subjects without a diagnosis of IPF, COPD, hypersensitivity pneumonitis, or pneumoconiosis. Control subjects were matched on a 1:1 basis with an IPF patient using patient age, gender and smoking status. Age matching required identification of control subjects born in the same year as IPF subjects, and who were alive at the age at which an IPF patient was first diagnosed. Comorbidities were examined in control subjects at the age when their matched patient was first diagnosed with IPF.

### Comorbidity definition

Comorbidities play a significant role in various health conditions and can greatly impact patient outcomes. In this study, we examined several comorbidities, including: hypertension, cardiac disease, cancer, atrial fibrillation, asthma, heart failure, diabetes, gastro-oesophageal reflux disease (GERD), cerebrovascular disease, hypothyroidism, chronic kidney disease, abdominal and peripheral vascular disease, pneumonia, hyperthyroidism, and dementia. In order to define and evaluate these comorbidities, we utilized read codes (medical codes) derived from GP records.

For each comorbidity, specific criteria were established to identify patients who met the comorbidity definition. We distinguished between prevalent and incident comorbidities, with equivalent dates examined in negative control subjects,. The earliest instance of meeting comorbidity criteria, which could be the diagnosis or history of diagnosis during a consultation in primary care, was defined as the first event date. We calculated the time interval between the onset of each comorbidity and the date of IPF or COPD diagnosis, to evaluate mortality relationships.

To facilitate data analysis, all comorbidities were classified as binary variables (yes = 1/no = 0) using pre-existing phenotypes developed and validated in the phenotype library available at CALIBER (https://phenotypes.healthdatagateway.org). The comorbidities were derived from read codes associated with each phenotype. While some comorbidities such as heart failure, hypertension, cerebrovascular disease, chronic kidney disease, diabetes, GERD, and pneumonia were derived from a single phenotype, others like atrial fibrillation, cardiac disease, and abdominal and peripheral vascular disease required combining multiple phenotypes. The library at CALIBER (https://www.caliberresearch.org) provided the necessary resources to develop and validate these phenotypes.

Atrial fibrillation, for instance, was defined as a binary variable using an existing phenotype (phenotype ID: PH1025), which combined evidence from various sources to define and date the phenotype. Cardiac disease was defined as a binary variable using nine existing phenotypes, encompassing conditions such as coronary heart disease, myocardial infarction, stable and unstable angina, and cardiac arrhythmia excluding atrial fibrillation. Abdominal and peripheral vascular disease was also classified as a binary variable, utilizing read codes and medical codes from two existing phenotypes: peripheral arterial disease (PH236) and abdominal aortic aneurysm (AAA) (PH34).

### Statistical methods

Data are given as means with standard deviations, or numbers of patients with percentages where appropriate. Median survival times and their respective 95% confidence intervals (95% CI) were determined using Kaplan–Meier curves with group differences assessed with the log-rank test. Follow up time represented the interval between the age of IPF or COPD diagnosis or the equivalent age in negative control subjects and the date of death or the last recorded date the subject was seen in the practice.

For the IPF cohort analysis, the primary outcome measure was the mortality risk for patients with a diagnosis of a prevalent comorbidity versus those with no prevalent comorbidity. In the case–control analysis, the primary outcome measure was the risk of death among patients with a relevant prevalent comorbidity compared to COPD patients and matched controls.

Factors that associated with time to death were determined using multivariable Cox proportional hazards regression models. Covariates adjusted for in Cox models included patient age, gender and smoking status (smoker, non-smoker) at the time of IPF or COPD diagnosis or the equivalent age in negative control subjects. Multivariable models examined the impact of individual comorbidities on patient survival, or the effect on survival of the cumulative number of comorbidities in an individual, coded as: no comorbidity identified (reference), 1-comorbidity, 2-comorbidities, 3-comorbidities, 4 + comorbidities, 5 + comorbidities. Schoenfeld residuals were used to check the proportional hazards assumption, martingale residual to assess nonlinearity, and deviance residuals (symmetric transformation of the martingale residuals) to examine influential observations. Missing values were not replaced nor extrapolated. *P*-values lower than 0.05 were considered statistically significant. Data analysis and data preparation was performed in R 3.6.9, using the R survival analysis packages: "survival" for computing survival analyses, "ggplot2", "survminer" and "forestplot" for visualizing survival analysis results.

## Results

### Baseline characteristics & comorbidity prevalence

Three thousand fifty-five IPF patients were included in the primary study cohort. These were matched 1:1 with COPD patients and the matched control cohort (Table 1). The mean age across all three cohorts was 71.5 years, and the studied population was predominantly male (66%). Smoking status (83%) and pack year smoking burden (median 10–12 pack years) was equivalent between IPF, COPD and matched control cohorts (Table 1). The median follow up time of 4.2 years for IPF patients (65% mortality during the study period) was significantly less than for COPD patients (6.2 years; 32% died during follow up) and the matched control population (7.9 years; 22% died during the study follow up period) (Table 1).

**Table 1:** Demographics and clinical characteristics table for prevalent comorbidity analysis in patients with idiopathic pulmonary fibrosis (IPF) or chronic obstructive pulmonary disease (COPD) matched for age, gender and smoking history, and control subjects without IPF or COPD, matched for age, gender and pack-year smoking history

**Characteristics**		**3,055 IPF patients**	**3,055 Matched COPD**	**3,055 Matched controls**		
**Age**	Mean age	71.5	71.5	71.5	IPF vs Matched COPD *P*-value:	IPF vs Matched controls *P*-value:
**Gender**	Male	2,003(66%)	2,003(66%)	2,003(66%)		
	Female	1,052 (34%)	1,052 (34%)	1,052 (34%)		
**Body Mass Index (BMI)**	Mean BMI, kg/m2	29.4	28.0	28.2		
	Not recorded	1,272 (42%)	1,279 (42%)	1,756 (57%)		
**Smoking history**	Non-smoker	512 (17%)	521 (17%)	512 (17%)		
	Ex-smoker	1,821 (60%)	1,800 (59%)	1,889 (62%)		
**Smoking currently**	Current Smoker	662 (22%)	734 (24%)	654 (21%)		
**Smokers**	Pack-year	11	12	10		
**Status**	Alive	1,056 (35%)	2,396 (68%)	2,092 (78%)		
	Dead	1,999 (65%)	659 (32%)	963 (22%)		
**Region England**	^*^North	913 (30%)	806 (29%)	889 (26%)		
	^*^South	2,142 (70%)	2,249 (71%)	2,166 (74%)		
**Overall follow-up time**	Median (IQR)	3.2 (1.3–6.0)	5.4 (2.3–9.2)	7.4 (4.1–11)	< 0.001	< 0.001
**Time to death**	Median (IQR)	2.6 (1.0–5.1)	4.3 (1.7–7.6)	5.5 (3.1–8.6)	< 0.001	< 0.001
**Follow-up time if alive**	Median (IQR)	4.5 (2.0–8.2)	6.0 (2.7–10.1)	7.9 (4.6–11.8)	< 0.001	< 0.001
**Comorbidity N(%)**	Hypertension	1,084 (35%)	1,266 (41%)	1,215 (40%)	< 0.001	< 0.001
	Cardiac Dx	826 (27%)	720 (24%)	659 (22%)	0.002	< 0.001
	Depression	689 (23%)	678 (22%)	500 (16%)	0.759	< 0.001
	Atrial Fibrillation	601 (20%)	509 (17%)	341 (11%)	0.003^*^	< 0.001
	Cancer	522 (17%)	622 (20%)	461 (15%)	0.001	0.037
	Asthma	448 (15%)	364 (12%)	129 (4%)	0.002	< 0.001
	Heart Failure	389 (13%)	164 (5%)	92 (3%)	< 0.001	< 0.001
	GERD	378 (12%)	280 (9%)	173 (6%)	< 0.001	< 0.001
	Diabetes	340 (11%)	319 (10%)	288 (9%)	0.409	0.032
	Chronic Kidney Dx	320 (10%)	207 (7%)	168 (5%)	< 0.001	< 0.001
	Cerebrovascular Dx	306 (10%)	299 (10%)	226 (7%)	0.797	< 0.001
	Hypothyroid	273 (9%)	168 (5%)	158 (5%)	< 0.001	< 0.001
	Pneumonia	257 (8%)	99 (3%)	36 (1%)	< 0.001	< 0.001
	Abdominal and Peripheral Vascular Dx	219 (7%)	195 (6%)	117 (4%)	0.242	< 0.001
	Bronchiectasis	145 (5%)	32 (1%)	4 (<1%)	< 0.001	< 0.001
	Hyperthyroid	57 (2%)	35 (1%)	32 (1%)	0.027	0.010
	Inflammatory Bowel Dx	55 (2%)	52 (2%)	23 (1%)	0.845	< 0.001
	Dementia	18 (1%)	73 (2%)	48 (2%)	< 0.001	< 0.001
	Sleep Apnoea	32 (1%)	19 (1%)	13 (< 1%)	0.092	0.007
	Liver Cirrhosis	24 (1%)	6 (< 1%)	2 (< 1%)	0.002	< 0.001
	Lung Cancer	23 (1%)	26 (1%)	2 (< 1%)	0.774	< 0.001
	Pulmonary Hypertension	1 (< 1%)	0	0		

Most prevalent comorbidities were significantly more common in IPF patients than COPD patients (Table 1). When compared to COPD patients, IPF patients were at increased risk of heart failure (Relative risk (RR) = 2.372), chronic kidney disease (RR = 1.546), gastro-oesophageal reflux disease (RR = 1.35), hypothyroidism (RR = 1.625) and pneumonia (RR = 2.596), as well as other common medical comorbidities (Supplementary Table 2). When compared to matched controls, IPF patients were at increased risk of prevalent: heart failure (RR = 4.228), asthma (RR = 3.473), GERD (RR = 2.185) and chronic kidney disease (RR = 1.905).

**Fig. 1:**
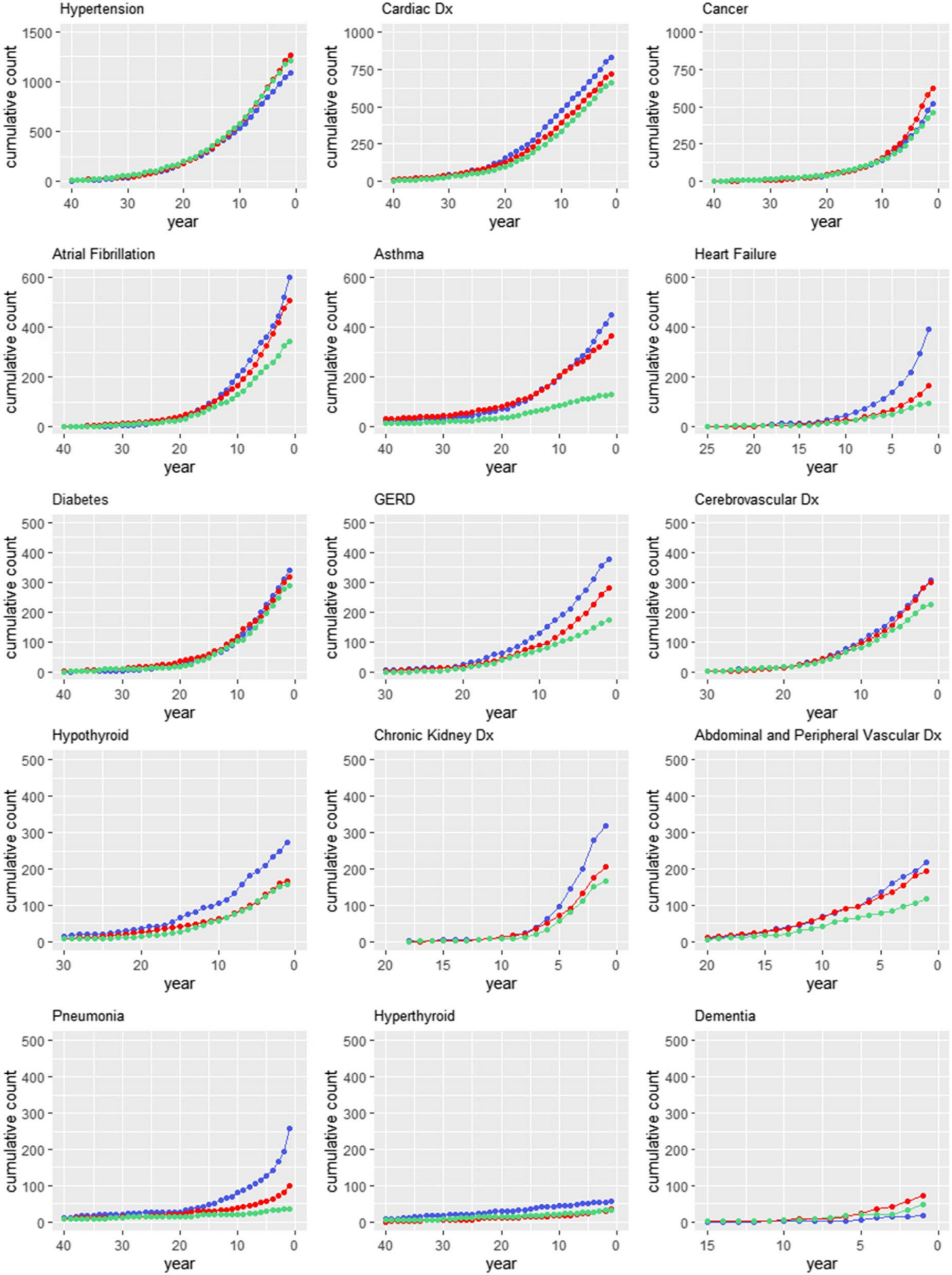
Graphs demonstrating the cumulative incidence of each of the fifteen selected prevalent comorbidities in patients with idiopathic pulmonary fibrosis (IPF; blue) or chronic obstructive pulmonary disease (COPD; red) matched for age, gender and smoking history, or control subjects (green) matched for age, gender and pack-year smoking history. The x-axis indicates years prior to IPF or COPD diagnosis or equivalent age in matched controls

When the date of onset of a comorbidity was examined, cardiac disease, GERD and hypothyroidism were seen to increase in incidence 20 years prior to IPF diagnosis when compared to COPD patients and matched controls despite similar smoking exposures. Heart failure and atrial fibrillation increased in incidence 10 years prior to IPF diagnosis while chronic kidney disease was identified with increased frequency 7 years prior to IPF diagnosis (Fig. 1).

### Individual comorbidities & survival

**Fig. 2:**
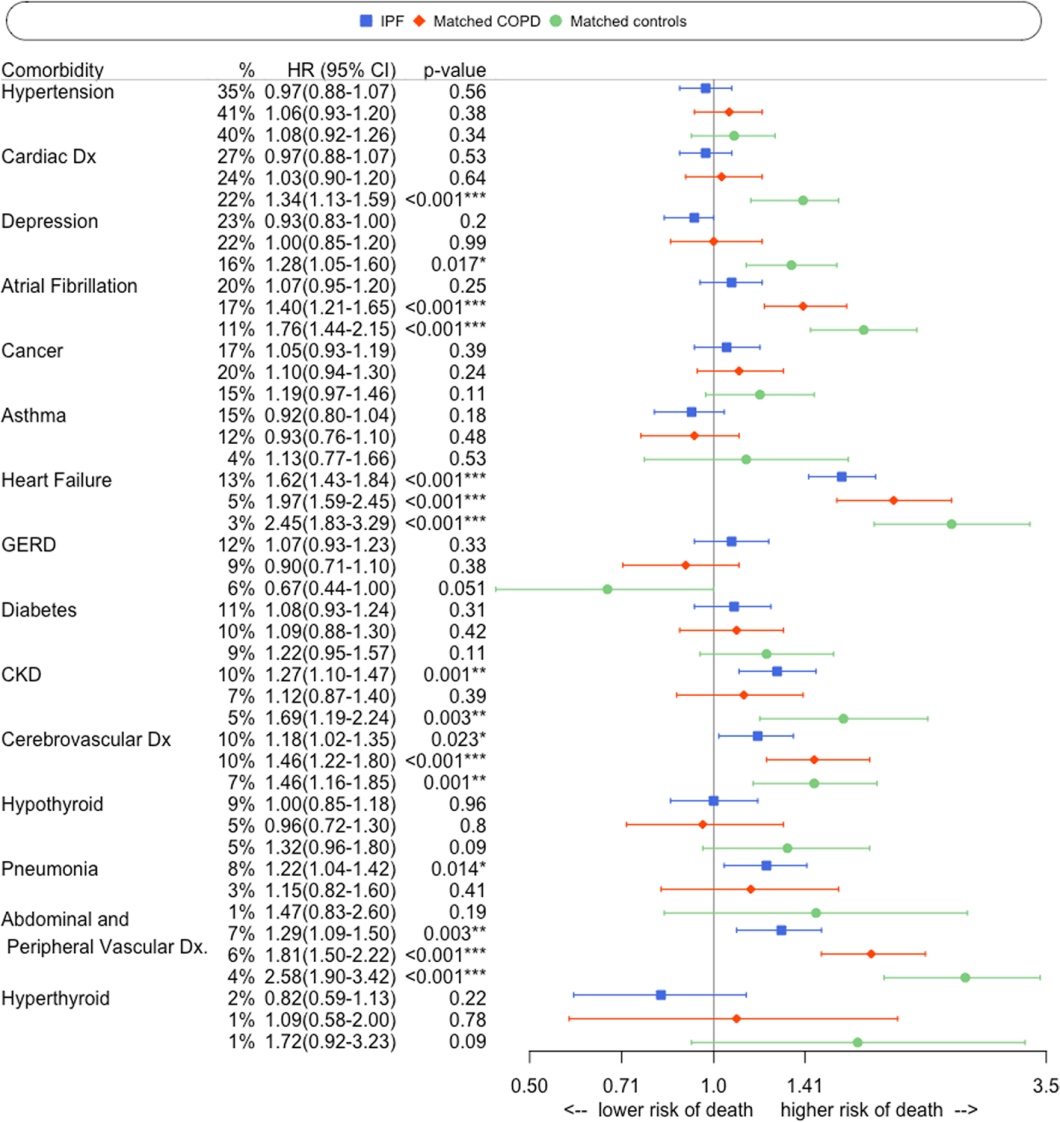
Multivariable Cox regression analyses displayed in a Forest Plot showing hazard ratios, 95% confidence intervals and *p*-values for the 15 most common comorbidities in IPF patients (*n* = 3885). IPF subjects are compared with COPD patients (*n* = 3885) and matched controls (*n* = 3885), with all models adjusted for patient age, gender and smoking status. IPF = idiopathic pulmonary fibrosis; COPD = chronic obstructive pulmonary disease

In IPF patients, five prevalent comorbidities were associated with reduced survival (Fig. 2): heart failure (Hazard ratio [HR] = 1.62, 95% Confidence Interval [CI]: 1.43–1.84, *p* < 0.001), chronic kidney disease (HR = 1.27, 95% CI: 1.10–1.47, *p* = 0.001), cerebrovascular disease (HR = 1.18, 95% CI: 1.02–1.35, *p* = 0.02), abdominal and peripheral vascular disease (HR = 1.29, 95% CI: 1.09–1.50, *p* = 0.003) and pneumonia (HR = 1.22, 95% CI: 1.04–1.42, *p* = 0.01). The only comorbidity that associated with reduced survival in COPD and not IPF was atrial fibrillation (HR = 1.40, 95% CI: 1.21–1.65, *p* < 0.001), whereas pneumonia and chronic kidney disease were not independently associated with mortality in COPD patients. Heart failure, cerebrovascular disease and abdominal and peripheral vascular disease consistently associated with reduced survival across all three study groups (Fig. 2).

### Cumulative comorbidities & survival

**Fig. 3:**
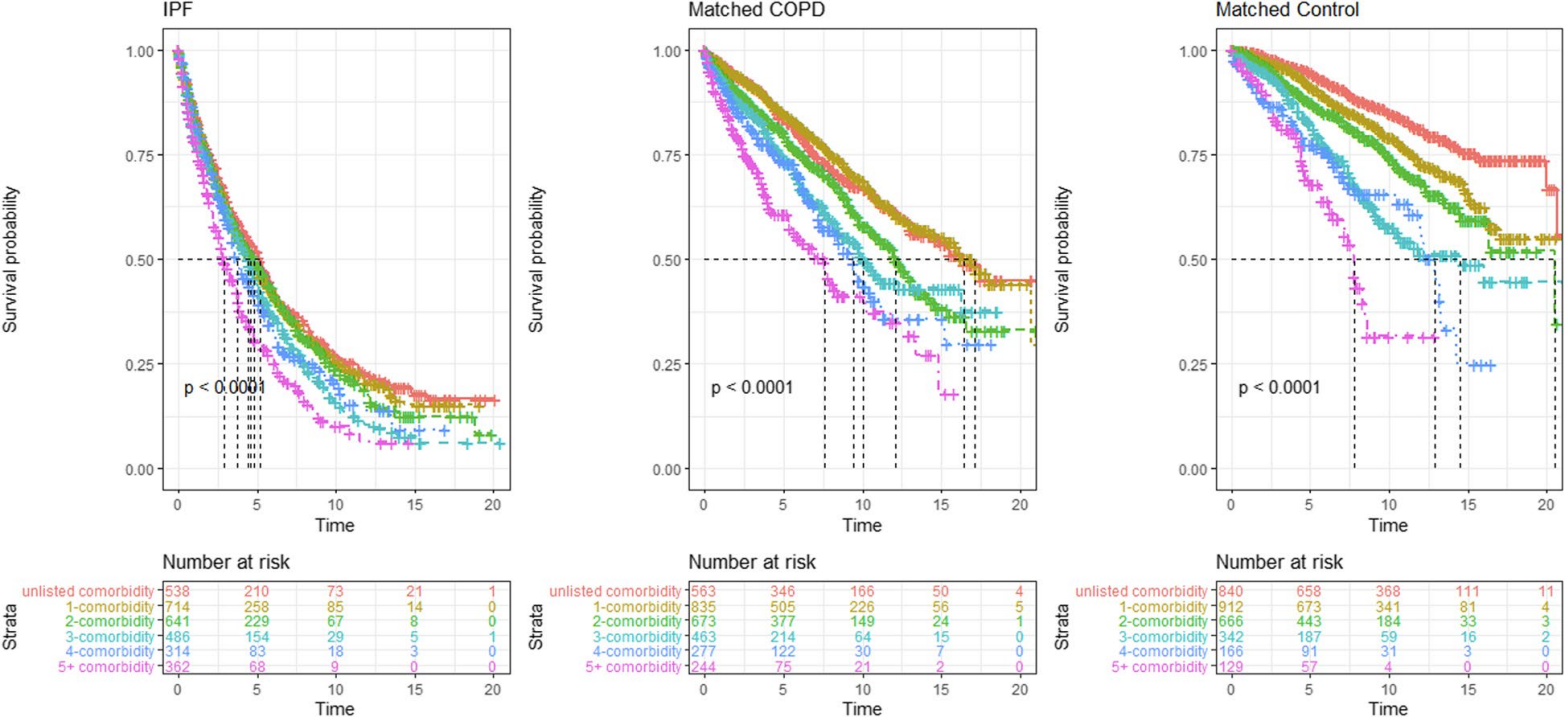
Kaplan Meier curves comparing survival in each of the three matched groups (IPF, COPD, Matched Controls) with increasing numbers of comorbidities. IPF = idiopathic pulmonary fibrosis; COPD = chronic obstructive pulmonary disease

A significant reduction in survival time (*p* < 0.0001) was observed in all cohorts as the number of comorbidities in an individual increased (Fig. 3). The reduced survival time seen with additional comorbidities was independent of patient age, gender and smoking status in all three patient groups (Supplementary Table 1).

**Fig. 4:**
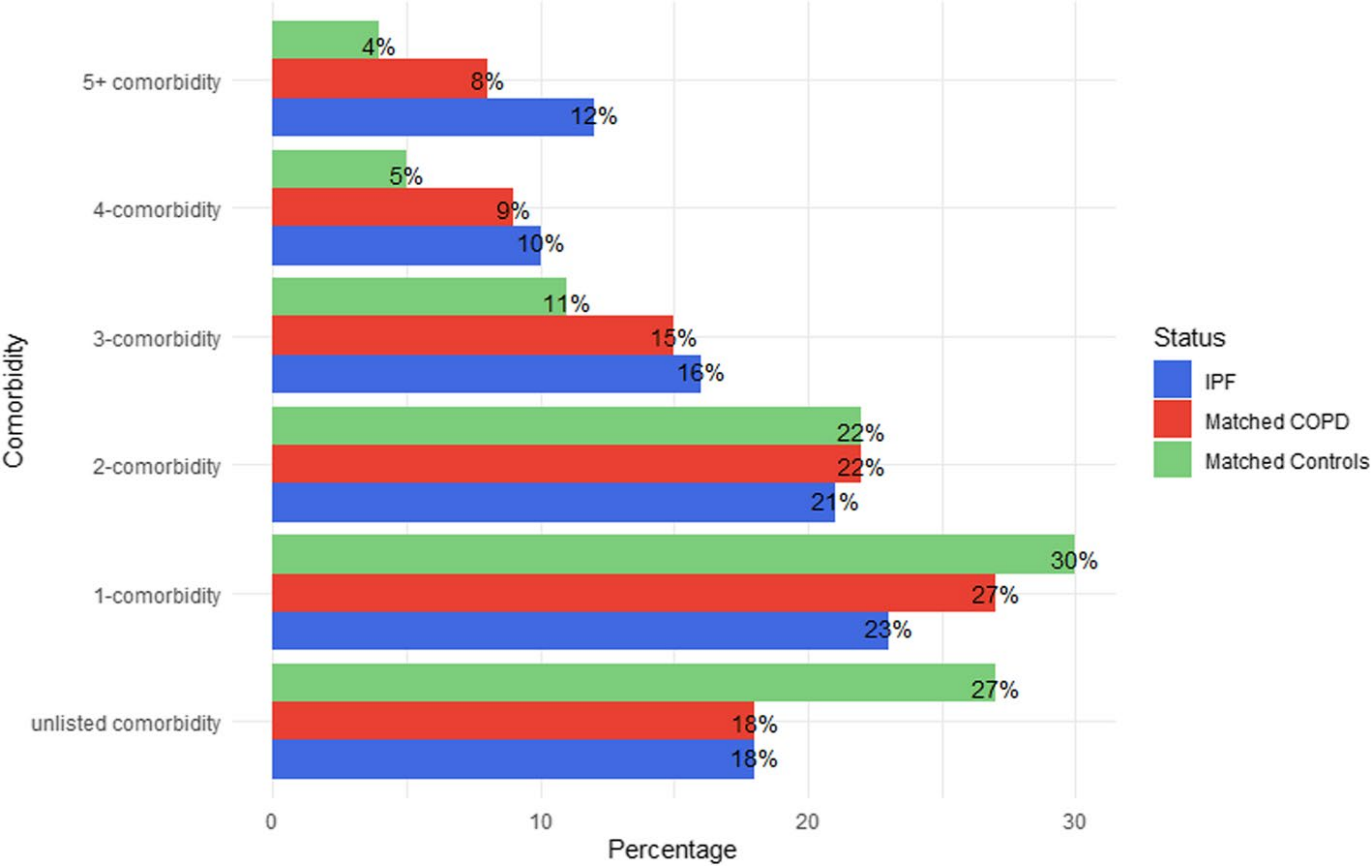
Numbers of subjects in each of the three matched groups (IPF, COPD, Matched Controls) with multiple comorbidities. IPF = idiopathic pulmonary fibrosis; COPD = chronic obstructive pulmonary disease

38% of IPF patients had 3 or more comorbidities prior to IPF diagnosis compared to 32% of COPD patients and 21% of age, gender, smoking-matched control subjects (Fig. 4). An IPF patient with no documented comorbidities at the time of IPF diagnosis had a median survival of 5 years which reduced with every additional comorbidity, so that having five or more comorbidities (11.8% of the population) resulted in a median survival of only 2.5 years (*p* < 0.0001).

### Individual comorbidities after IPF diagnosis

Though IPF patients lived on average only two-thirds as long as COPD patients after diagnosis, 8 of the 14 most common incident comorbidities (cardiac disease, atrial fibrillation, heart failure, GERD, diabetes, hypothyroidism, pneumonia, bronchiectasis) were at least as common in IPF patients as they were in COPD patients (Table 2). Incident cardiac disease, heart failure and pneumonia were more frequently in IPF patients than in the matched control population (*p* < 0.001). Despite the shorter follow up time, almost twice as many IPF patients developed pneumonia when compared to COPD patients (Table 2), and this was almost five times as many cases of pneumonia as were seen in the matched control cohort during follow up (*p* < 0.001).

**Table 2:** Primary care defined incident comorbidities identified in each of the three study groups following the idiopathic pulmonary fibrosis (IPF) or chronic obstructive pulmonary disease (COPD) diagnosis date, or equivalent age in matched controls

Comorbidity	3,055 IPF patients	3,055 Matched COPD	3,055 Matched controls	IPF vs Matched COPD *P*-value:	IPF vs Matched Controls* P*-value:
*Hypertension*	7%	10%	14%	< 0.001	< 0.001
*Cardiac Dx*	12%	11%	8%	0.373	< 0.001
*Atrial Fibrillation*	12%	11%	11%	0.523	0.227
*Cancer*	17%	20%	20%	0.002	0.001
*Asthma*	2%	4%	1%	< 0.001	0.005
*Heart Failure*	8%	8%	5%	1.000	< 0.001
*GERD*	6%	6%	5%	0.589	0.051
*Diabetes*	7%	7%	8%	0.618	0.027
*Chronic Kidney Dx*	11%	15%	16%	< 0.001	< 0.001
*Cerebrovascular Dx*	5%	7%	8%	< 0.001	< 0.001
*Hypothyroid*	2%	2%	2%	1.000	0.675
*Pneumonia*	9%	5%	2%	< 0.001	< 0.001
*Abdominal and Peripheral Vascular Dx*	3%	4%	4%	0.033	0.166
*Bronchiectasis*	3%	1%	< 1%	< 0.001	< 0.001
*Hyperthyroid*	< 1%	< 1%	< 1%	1.000	1.000
*Inflammatory Bowel Dx*	1%	< 1%	1%	0.229	1.000
*Dementia*	3%	5%	7%	< 0.001	< 0.001
*Sleep Apnoea*	1%	1%	< 1%	0.210	0.005
*Liver Cirrhosis*	< 1%	< 1%	< 1%	0.080	0.307
*Lung Cancer*	3%	2%	1%	< 0.001	< 0.001
*Pulmonary Hypertension*	< 1%	< 1%	< 1%	-	0.070

## Discussion

Our study findings highlight five important findings in patients with IPF. First, the high incidence of pre-index multiorgan disease in IPF suggests that rather than a respiratory workup alone, at diagnosis, IPF patients should routinely be worked up for multiorgan disease. Second, the increased incidence of multiorgan disease in IPF cannot be attributed solely to the deleterious effects of smoking as the number of IPF patients with three or more comorbidities was almost double that seen in age, gender and smoking matched controls. Third, several comorbidities (cardiac disease, atrial fibrillation, heart failure, hypothyroidism, GERD, chronic renal failure) showed increased incidence in IPF compared to COPD, 7–10 years before the date of IPF/COPD diagnosis suggesting that mechanisms other than chronic systemic inflammation should be examined to explain increased IPF multimorbidity. Fourth, comorbidities in IPF are clinically important. Prevalent heart failure, chronic renal failure, cerebrovascular disease and abdominal and peripheral vascular disease, all independently associated with mortality, suggesting that IPF patients die from, rather than with these comorbidities. Lastly, incident comorbidities were comparable between IPF and COPD patients despite COPD subjects having a significantly longer follow up period. As disease mechanisms resulting in multiorgan damage appear to propagate through the disease course of IPF, regular routine patient assessment should therefore consider multiorgan evaluation.

The burden of comorbidity in patients with IPF has not been studied as extensively as it has in COPD patients. Estimates of comorbidity prevalence vary widely between studies, but the rates of multiple prevalent comorbidity identified in our study are in line with those documented in prior registry studies [9, 27]. Regarding individual comorbidities, previous studies have generated comparable estimates of the prevalence of hypertension [9, 28], cardiac disease [9, 27], thyroid disease [27], GERD [9], cerebrovascular disease [10], heart failure [10] in IPF populations (Table 1). The prevalence of diabetes was lower in our IPF cohort than prior studies (11% versus 23–45%) [9, 10, 29], but depression was more commonly seen in our study than in prior reports (23% versus 3–15%) [9, 10, 29]. Mendelian randomisation has demonstrated causal associations between hypothyroidism and GERD and the development of IPF in a prior study [30]. This observation aligns with our study where these comorbidities demonstrated increased incidence in subjects who later developed IPF. Conversely however, mendelian randomisation demonstrated that IPF has causal associations with a reduced risk of hypertension [30]. These findings are again supported by our study where hypertension was the only comorbidity more common in the matched control population than IPF subjects. Dementia was found to be less prevalent in the IPF cohort compared to the COPD and matched control populations. This may be an artefact of the small number of overall cases with dementia identified across all three study groups which could lead to spurious statistically significant differences. The limited extent of screening for dementia in the general population during the timeframe of data collection for this study also cautions us to interpret this finding with care.

Comorbidity has been more comprehensively studied in COPD. By matching pack-year smoking histories between IPF and COPD, our COPD population had a lower pack-year smoking exposure than might be seen in commonly reported COPD populations. Nevertheless, despite this reduced smoking burden in our COPD cohort, our estimates for comorbidity prevalence in COPD are similar to those previously published for general COPD cohorts. The prevalence of hypertension in COPD patients reported in the literature ranges from 30 to 52% [24, 25], in keeping with the 41% prevalence we report. Similarly, published prevalence estimates for cardiovascular disease [25, 31, 32], chronic kidney disease [24, 33], diabetes mellitus [24, 25] and heart failure [25] are all comparable to the results we report in the study (Table 1). This concordance between previously published comorbidity estimates in COPD and the estimates we report strengthens the likelihood that our estimates for comorbidity in IPF reflect real-world comorbidity prevalence.

The excess multiorgan comorbidities identified in COPD patients have been ascribed to the occurrence of chronic systemic inflammation, highlighted by the persistently raised levels of inflammatory mediators in peripheral blood [34]. This chronic inflammation can lead to widespread vascular endothelial dysfunction and in turn can result in cardiovascular, renal, and cerebrovascular end-organ damage [35]. IPF is not characteristically considered a disease of chronic inflammation, primarily due to the poor (at times, deleterious) effect of immunosuppressive therapies in the treatment of IPF [35, 36]. However, the impact of endothelial dysfunction in the aetiology of IPF [37–39] has received increased attention in recent years and has been speculated as a potential cause for multiorgan disease.

It is possible that a genetic predisposition to vascular endothelial dysfunction may explain the high incidence of cardiac, cerebrovascular, renal, and abdominal and peripheral vascular disease seen in a subset of our IPF population. Microvascular abnormalities are common in IPF, with fibrotic areas being far less vascularised than non-fibrotic areas. This abnormal vascularization in IPF may well inhibit normal repair mechanisms or conversely, be a compensatory mechanism that limits fibrogenesis [40] and abets the theory of IPF as a disease of abnormal wound healing in response to repeated injury of alveolar epithelial cells [41]. The pathogenesis of IPF also involves aberrant local immune responses [42], which may influence the systemic immune landscape and thus contribute to multimorbidity.

The identification of excess telomere shortening and cellular senescence [43] in pulmonary fibrosis has also raised the possibility that IPF represents a pulmonary manifestation of premature ageing. Large volumes of senescent cells have been found to accumulate in IPF lungs [44], but have only shown a limited presence in COPD lungs [45]. Type II alveolar epithelial cells (AECs) have been shown to have short telomeres [46] and to uniquely express senescence markers in IPF [47]. These findings have in turn been shown to lead to age-dependent lung remodelling and fibrosis in mouse models [48]. Lastly, telomere length in peripheral blood leukocytes has been shown to predict survival in IPF patients [49]. Multisystem ageing in a subset of IPF patients could therefore explain the increased levels of comorbidity observed in our study when compared to COPD or matched control subjects.

There were limitations to this study. The diagnosis of IPF relied on electronic health record codes which could have been misclassified by general practitioners. However, we believe miscoding to be limited at best, as survival in our IPF cohort corresponds well with expected life expectancy over the period in which data was collected. Similarly, identification of comorbidities relied on individuals seeking health care services and GPs undertaking relevant tests. It is possible that patients who avoided health services or testing could have been assigned as having no comorbidities. Our prevalent comorbidity rate may therefore have been an underestimate. We were not able to assess data on IPF disease severity (lung function, imaging), hospital or intensive care admission data, or antifibrotic treatment in our population as the study period covered the spectrum of pre-IPF diagnostic guidelines and changes to diagnostic guidelines. We were also unable to access data on the severity of individual comorbidities or their mode of diagnosis but we do not believe these factors would have influenced our estimates of prevalent comorbidities. It is possible that IPF patients with large numbers of comorbidities might not have been as aggressively managed in a hospital setting (regarding intubation and ventilation) when compared to patients with COPD and similar numbers of comorbidities. This in turn could account for some of the impact of multiple comorbidities on survival identified in our IPF patients. The lower pack-year smoking history in our COPD population is notable when compared to more general COPD populations described in the literature. Nevertheless, the fact that comorbidity prevalence rates identified in our study are in keeping with prior reports of comorbidity prevalence in general COPD cohorts suggests that the lower pack-year history in our data may not have influenced our results unduly. Notably, we did not exclude patients with connective tissue disease (CTD) from the COPD or matched control cohorts. CTDs are relatively common, and only a minority have associated fibrosing lung disease. Excluding CTD patients, who often have multi-system disease, might have strongly biased our findings against the presence of comorbidities in the control population. Therefore, when considering that patients with CTD were present in the negative control cohort, our findings of increased comorbidity in the IPF cohort would appear to be strengthened. A final limitation of the study design and analysis is the potential measurement error in variables such as smoking pack-year history that may introduce a degree of residual confounding, as well as the lack of recording of BMI in almost 50% of cases across the three cohorts.

In conclusion our study shows the excess burden of prevalent comorbidities seen in IPF which is not explained by smoking-related damage and is greater than that seen in COPD where systemic inflammation is thought to drive multiorgan disease. The mortality impact of these comorbidities emphasizes the need to consider multiorgan mechanisms of injury in patients that develop IPF.

### Supplementary Information

Supplementary Material 1: Supplementary Figure 1. Flow diagram showing the study population and control groups. CPRD=clinical practice research datalink, IPF=idiopathic pulmonary fibrosis, COPD=chronic obstructive pulmonary disease; EAA=extrinsic allergic alveolitis.

Supplementary Material 2: Supplementary Table 1. Multivariable Cox regression analyses showing the association with mortality for cumulative comorbidities (expressed as an ordinal variable categorised as 0, 1, 2, 3, 4, or 5+) diagnosed prior to idiopathic pulmonary fibrosis (IPF) and chronic obstructive pulmonary disease (COPD). CI=confidence intervals (CI). Supplementary Table 2. Risk ratios and confidence intervals (CI) for prevalent comorbidities in the three study groups: idiopathic pulmonary fibrosis (IPF), matched chronic obstructive pulmonary disease (COPD) and age, gender and pack-year smoking matched controls.; RR = Relative Risk (Risk Ratio).

## Data Availability

No datasets were generated or analysed during the current study.
