# Supplementary material for: Delineating excess comorbidities in idiopathic pulmonary fibrosis: an observational study
Source: Respir Res. 2024 Jun 19;25:249. doi: 10.1186/s12931-024-02875-2 (PMC11186192; doi:10.1186/s12931-024-02875-2)
Supplement: Supplementary file 1 — Supplementary Material 1: Supplementary Figure 1. Flow diagram showing the study population and control groups. CPRD=clinical practice research datalink, IPF=idiopathic pulmonary fibrosis, COPD=chronic obstructive pulmonary disease; EAA=extrinsic allergic alveolitis. [file 12931_2024_2875_MOESM1_ESM.docx]

**SUPPLEMENTARY FIGURES:**

**
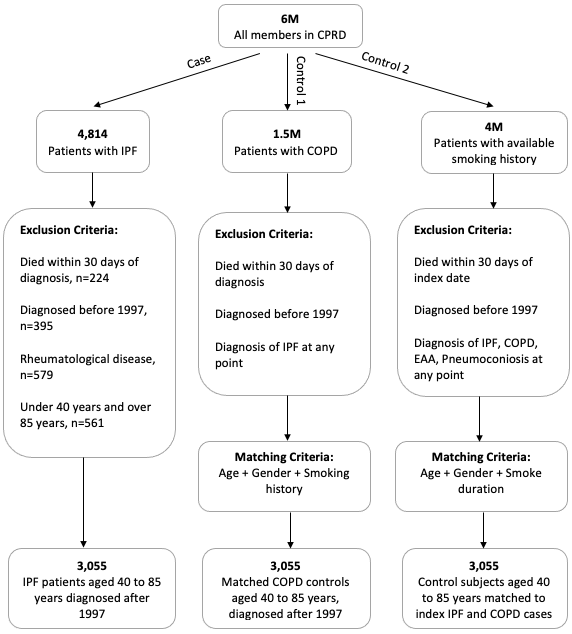
**

Supplementary Figure 1: Flow diagram showing the study population and control groups. CPRD=clinical practice research datalink, IPF=idiopathic pulmonary fibrosis, COPD=chronic obstructive pulmonary disease; EAA=extrinsic allergic alveolitis
