# Supplementary material for: Delineating excess comorbidities in idiopathic pulmonary fibrosis: an observational study
Source: Respir Res. 2024 Jun 19;25:249. doi: 10.1186/s12931-024-02875-2 (PMC11186192; doi:10.1186/s12931-024-02875-2)
Supplement: Supplementary file 2 — Supplementary Material 2: Supplementary Table 1. Multivariable Cox regression analyses showing the association with mortality for cumulative comorbidities (expressed as an ordinal variable categorised as 0, 1, 2, 3, 4, or 5+) diagnosed prior to idiopathic pulmonary fibrosis (IPF) and chronic obstructive pulmonary disease (COPD). CI=confidence intervals (CI). Supplementary Table 2. Risk ratios and confidence intervals (CI) for prevalent comorbidities in the three study groups: idiopathic pulmonary fibrosis (IPF), matched chronic obstructive pulmonary disease (COPD) and age, gender and pack-year smoking matched controls.; RR = Relative Risk (Risk Ratio). [file 12931_2024_2875_MOESM2_ESM.docx]

Supplementary Table 1: Multivariable Cox regression analyses showing the association with mortality for cumulative comorbidities (expressed as an ordinal variable categorised as 0, 1, 2, 3, 4, or 5+) diagnosed prior to idiopathic pulmonary fibrosis (IPF) and chronic obstructive pulmonary disease (COPD). CI=confidence intervals (CI).

| Groups | Variables | Hazard Ratio | %95 CI | P-value |
| --- | --- | --- | --- | --- |
| IPF | Age (years) | 1.05 | 1.04 – 1.05 | <0.001* |
|  | Female | 0.71 | 0.64 – 0.78 | <0.001* |
|  | Smoker  (never vs ever) | 1.31 | 1.15 – 1.49 | <0.001* |
|  | Comorbidity  number | 1.04 | 1.01 – 1.07 | 0.009* |
| Matched COPD | Age (years) | 1.10 | 1.09 – 1.11 | <0.001* |
|  | Female | 0.77 | 0.67 – 0.89 | <0.001* |
|  | Smoker  (never vs ever) | 1.44 | 1.18 – 1.76 | <0.001* |
|  | Comorbidity number | 1.11 | 1.06 – 1.16 | <0.001* |
| Matched Controls | Age (years) | 1.10 | 1.08 – 1.11 | <0.001* |
|  | Female | 0.69 | 0.58 – 0.82 | <0.001* |
|  | Smoker  (never vs ever) | 1.26 | 0.99 – 1.60 | 0.064 |
|  | Comorbidity  number | 1.23 | 1.16 – 1.30 | <0.001* |
